# Synthesis, structure determination and characterization by UV–Vis and IR spectroscopy of bis­(diiso­propyl­ammonium) *cis*-di­chlorido­bis(oxalato-κ^2^ *O* ^1^,*O* ^2^)stannate(IV)

**DOI:** 10.1107/S2056989019006030

**Published:** 2019-05-03

**Authors:** Bougar Sarr, Abdou Mbaye, Cheikh Abdoul Khadir Diop, Mamadou Sidibe, Yoann Rousselin

**Affiliations:** a Laboratoire de Chimie Minérale et Analytique, Département de Chimie, Faculté des Sciences et Téchniques, Université Cheikh Anta Diop, Dakar, Senegal; b Laboratoire de Chimie et de Physique des Matériaux (LCPM) de l’Université Assane Seck de Ziguinchor (UASZ), BP 523 Ziguinchor, Senegal; c ICMUB-UMR 6302, 9, avenue Alain Savary 21000 Dijon, France

**Keywords:** crystal structure, tin(IV) oxalate derivative, spectroscopic studies, hydrogen bonding

## Abstract

The organic–inorganic title salt, (C_6_H_16_N)_2_[Sn(C_2_O_4_)_2_Cl_2_] or (^*i*^Pr_2_NH_2_)_2_[Sn(C_2_O_4_)_2_Cl_2_], was obtained by reacting bis­(diiso­propyl­ammonium) oxalate with tin(IV) chloride dihydrate in methanol. The Sn^IV^ atom is coordinated by two chelating oxalate ligands and two chloride ions in *cis* positions, giving rise to an [Sn(C_2_O_4_)_2_Cl_2_]^2−^ anion (point group symmetry 2), with the Sn^IV^ atom in a slightly distorted octa­hedral coordination. The cohesion of the crystal structure is ensured by the formation of N—H⋯O hydrogen bonding between (^i^Pr_2_NH_2_)^+^ cations and [SnCl_2_(C_2_O_4_)_2_]^2−^ anions. This gives rise to an infinite chain structure extending parallel to [101]. The main inter-chain inter­actions are van der Waals forces. The electronic spectrum of the title compound displays only one high intensity band in the UV region assignable to ligand–metal ion charge-transfer (LMCT) transitions. An IR spectrum was also recorded and is discussed.

## Abstract 2

In the crystal structure of the title compound, (C_6_H_16_N)_2_[Sn(C_2_O_4_)_2_Cl_2_], the cations are linked to the anions by N—H⋯O hydrogen bonds to generate chains along the *c*-axis direction. Only van der Waals inter­actions are observed between the chains.

## Chemical context   

As a result of their numerous applications in medicine, industry and agriculture (Kapoor *et al.*, 2005 ▸), tin(IV) carboxyl­ate compounds have attracted the attention of several research groups, resulting in the preparation and characterization of new compounds (Christie *et al.*, 1979 ▸; Ng & Kumar Das, 1993 ▸; Rocamora-Reverte *et al.*, 2012 ▸; Reichelt & Reuter, 2014 ▸). Derivatives of tin(IV) oxalate are a subclass of the tin(IV) carboxyl­ate family and have likewise been studied extensively because oxalate ions, C_2_O_4_^2–^, play an important role as counter-ions or complex ligands in inorganic as well as in organometallic chemistry. One of the motivations to study these compounds is related to the rich coordinating behaviour of the oxalato ligand, which can adopt a monodentate, a bridging monodentate, a bridging bidentate, a monochelating, bidentate or a bichelating mode (Miskelly *et al.*, 1983 ▸; Sow *et al.*, 2012 ▸; Świtlicka-Olszewska *et al.*, 2014 ▸). In this context, our group has previously published the syntheses and crystal structure determinations of some tin(IV) oxalate derivatives (Sarr *et al.*, 2013 ▸, 2018 ▸). As a continuation of this work, we have studied the inter­action between bis­(diiso­propyl­ammonium) oxalate with tin(IV) chloride dihydrate, which yielded the title salt (C_6_H_16_N)_2_[Sn(C_2_O_4_)_2_Cl_2_] or (^*i*^Pr_2_NH_2_)_2_[Sn(C_2_O_4_)_2_Cl_2_]. Its crystal structure was determined by single crystal X-ray diffraction and was confirmed by infrared and UV–visible spectroscopic studies.
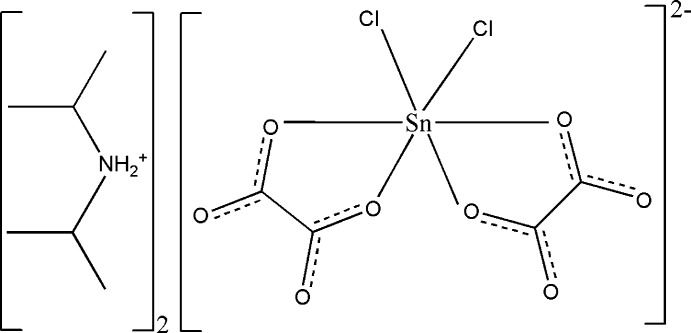


## Structural commentary   

**Figure 1:**
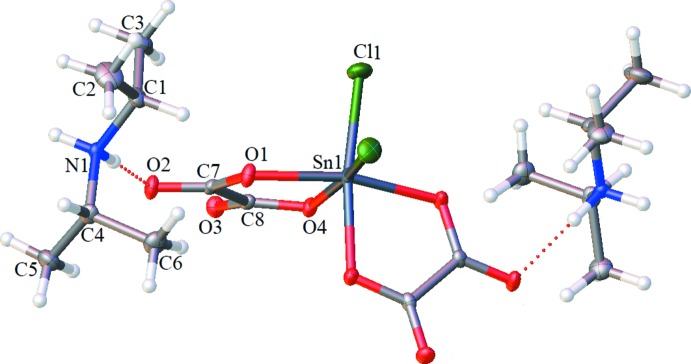
The mol­ecular entities in the organic–inorganic title salt drawn with displacement ellipsoids at the 50% probability level; hydrogen atoms are depicted as spheres of arbitrary radius

The asymmetric unit of (^*i*^Pr_2_NH_2_)_2_[Sn(C_2_O_4_)_2_Cl_2_] comprises one diiso­propyl­ammonium cation and one half of an [Sn(C_2_O_4_)_2_Cl_2_]^2−^ anion, the other half being completed by the application of twofold rotation symmetry (Fig. 1 ▸) with the rotation axis running through the central Sn^IV^ atom. The latter is chelated by two oxalate ligands and is additionally ligated by two Cl atoms in *cis* positions within a distorted octa­hedral coordination sphere [Cl1^i^—Sn1—Cl1= 97.26 (4)°; O1—Sn1—O4 = O1^i^—Sn1—O4^i^ = 79.27 (6) °, O1—Sn1—O4^i^ = 90.21 (6)°; symmetry code: (i) *x* + 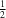, −*y* + 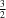, *z* − 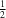]. Atoms Cl1^i^, O1, O1^i^ and O4 define the equatorial plane [with a slight shift of Sn1 from this plane by 0.1163 (5) Å; rms = 0.0687 Å] while Cl1 and O4^i^ occupy the axial positions. The O4^i^—Sn1—Cl1 angle of 168.50 (4)° indicates a considerable deviation from linearity, which might be explained by the difference in size of the Cl and O atoms and by the small bite angle of 79.27 (6)° between the central Sn^IV^ atom and the chelating O1 and O4 atoms.

As in the related structure of (^*i*^Pr_2_NH_2_)_2_[Sn(C_2_O_4_)_2_I_2_] (Sarr *et al.*, 2018 ▸), the lengths of the C—O bonds within the oxalate ligands vary slightly because of the different functions of the oxygen atoms involved in the coordination of Sn^IV^. The C—O bond lengths of coordinating O atoms [O1—C7 = 1.289 (2) Å; O4—C8 = 1.289 (2) Å] are significantly longer than those of non-coordinating O atoms [O2—C7 = 1.222 (2) Å, O3—C8 = 1.223 (2) Å]. The Sn1—C1l distance of 2.3422 (9) Å as well as the Sn—O distances of 2.0710 (16) Å (O1) and of 2.1057 (15) Å (O4) are slightly shorter than corresponding bonds reported previously (Reichelt & Reuter, 2014 ▸; Sarr *et al.*, 2013 ▸; Diop *et al.*, 2011 ▸; Sow *et al.*, 2013 ▸; Skapski *et al.*, 1974 ▸).

## Supra­molecular features   

**Figure 2:**
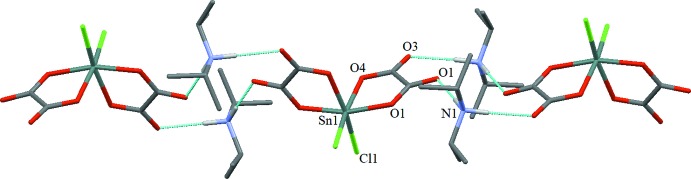
An infinite chain in the title structure, showing the N—H⋯O hydrogen bonds as light-blue dashed lines. C-bound H atoms have been omitted for clarity.

**Figure 3:**
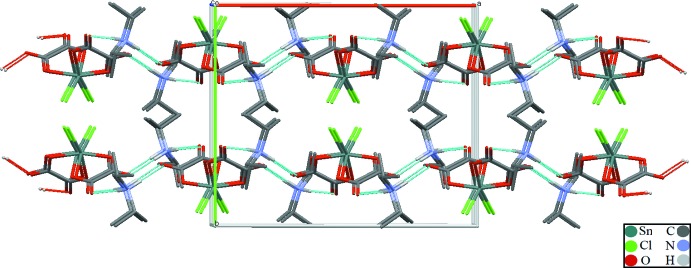
The crystal packing of the title compound in a view down [001]. C-bound H atoms have been omitted for clarity.

Each anionic complex [Sn(C_2_O_4_)_2_Cl_2_]^2–^ is linked to two neighbours *via* four diiso­propyl­ammonium cations through N—H⋯O hydrogen bonds, leading to infinite chains parallel to [101] (Table 1 ▸, Fig. 2 ▸). In a chain, the two non-coordinating oxygen atoms (O2 and O3) of each oxalate ligand are involved as acceptors in hydrogen-bonding inter­actions (Table 1 ▸). The chains are arranged into layers extending parallel to (010), mainly inter­connected by van der Waals forces (Fig. 3 ▸).

## Database survey   

A search in the Cambridge Structural Database (CSD, version 5.40, update Nov. 2018; Groom *et al.*, 2016 ▸) resulted in 226 hits dealing with diiso­propyl­ammonium cations while only one hit deals with the [Sn(C_2_O_4_)_2_Cl_2_]^2–^ anion (Sarr *et al.*, 2013 ▸).

## Synthesis and crystallization   

The title salt was obtained by mixing bis­(diiso­propyl­ammonium) oxalate (^*i*^Pr_2_NH_2_)_2_C_2_O_4;_ 0.30 g; 15.50 mmol) and tin(IV) chloride dihydrate (SnCl_2_·2H_2_O; (0.34 g; 15.50 mmol) in a 1:1 molar ratio in methanol. The obtained yellow solution was stirred for one h and then filtered. Colourless prism-like crystals were obtained by slow evaporation of the filtrate over a period of ten days.

**Figure 4:**
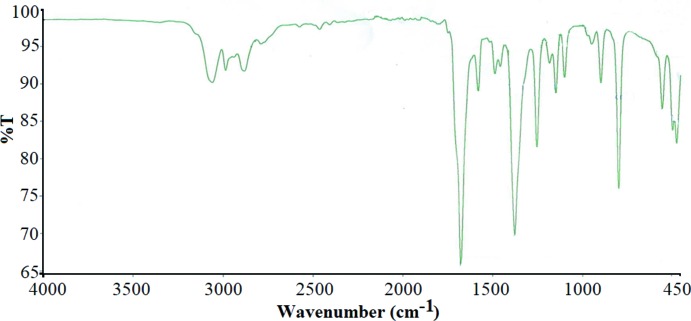
The IR spectrum of the title compound.

The IR spectrum confirms the presence of oxalate and diiso­propyl­ammonium groups in the title salt. In addition, the appearance of valence vibrations (–CO_2_) in the form of three bands shows that the oxalate ligands are not centrosymmetric, in agreement with the difference in the C—O bond lengths revealed by the X-ray study. Attributions of the vibrational bands of the title compound were made by comparison with previous studies (Sarr *et al.*, 2018 ▸; Marinescu *et al.*, 2002 ▸; Li *et al.*, 2008 ▸). The vibrational bands at 3061 and 1579 cm^−1^ in the IR spectrum (Fig. 4 ▸) are assigned to the stretching and deformation modes ν(N—H) and δ(N—H), respectively, of –NH_2_– in the ammonium group. The bands at 1675, 1375 and 1251 cm^−1^ are attributed to the asymmetric and symmetric vibrations of the oxalate –CO_2_ moiety while that at 795 cm^−1^ corresponds to the deformation vibrations δ(C—O) (Sarr *et al.*, 2018 ▸; Marinescu *et al.*, 2002 ▸; Li *et al.*, 2008 ▸). The frequencies of stretching vibrations of the oxalate group often show slight deviations owing to the different coordination modes. The bands at 2882 cm^−1^ are assigned to the valence vibrations ν(C—H) and those at 1486 cm^−1^ to the deformation vibrations δ(C—H).

**Figure 5:**
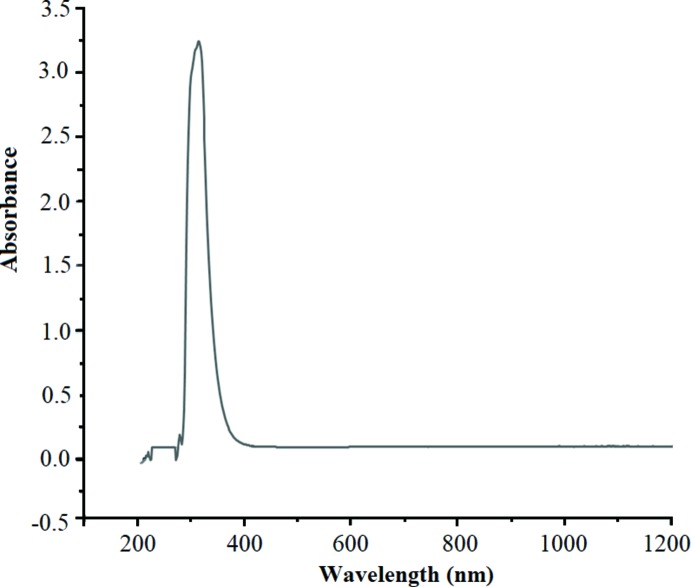
The electronic spectrum of the title compound.

The electronic spectrum of the title compound is shown in Fig. 5 ▸. In the ultraviolet region, only one strong absorption band with a shoulder is observed. Generally, only π→π*, n→π* and LMCT transitions can be observed in the ultraviolet–visible (UV–Vis) region. The σ→σ* transition requires an absorption of a photon with a wavelength which does not fall in the UV–Vis range. Thus, this strong absorption band at 320 nm (Fig. 5 ▸) may be assigned to ligand-metal ion charge transfer (LMCT) (Kane *et al.*, 2016 ▸). However, as the ligand-based π→π* / n→π* transitions absorb in the same area as the LMCT transitions, we cannot exclude a possibility of superposition of these transitions (ligand-based and LMCT) owing to the form of the absorption band (Ford & Vogler, 1993 ▸; Filho *et al.*, 2000 ▸). In the title compound, the Sn^IV^ atom is surrounded by electron rich ligands (chlorido and oxalato), and π→π* and n→π* transitions may result from the non-binding electron pairs present on chlorine atoms or oxygen atoms of oxalate (Wojciechowska *et al.*, 2016 ▸).

## Refinement details   

Crystal data, data collection and structure refinement details are summarized in Table 2 ▸. All H atoms were placed in geometrically idealized positions and constrained to ride on their parent atoms, with a N—H distance of 0.91 Å, C_meth­yl_—H = 0.98 Å and C_methine_ = 1.0 Å, and with *U*_iso_(H) = 1.2*U*_eq_ (C,N) or 1.5*U*_eq_(C_meth­yl_). Three reflections were omitted from refinement because they were obstructed by the beam stop.

## Supplementary Material

Crystal structure: contains datablock(s) I. DOI: 10.1107/S2056989019006030/wm5500sup1.cif

Structure factors: contains datablock(s) I. DOI: 10.1107/S2056989019006030/wm5500Isup2.hkl

CCDC reference: 1833609

Additional supporting information:  crystallographic information; 3D view; checkCIF report

## Figures and Tables

**Table 1:** Hydrogen-bond geometry (Å, °)

*D*—H⋯*A*	*D*—H	H⋯*A*	*D*⋯*A*	*D*—H⋯*A*
N1—H1*A*⋯O3^i^	0.91	2.05	2.943 (2)	167
N1—H1*B*⋯O2^ii^	0.91	1.95	2.855 (2)	177

Symmetry codes: (i) 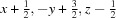; (ii) 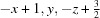.

**Table 2:** Experimental details

Crystal data	
Chemical formula	(C_6_H_16_N)_2_[Sn(C_2_ClO_4_)_2_]
*M*_r_	570.02
Crystal system, space group	Monoclinic, *C*2/*c*
Temperature (K)	100
*a*, *b*, *c* (Å)	16.275 (8), 13.581 (6), 11.116 (4)
β (°)	98.40 (3)
*V* (Å^3^)	2430.7 (18)
*Z*	4
Radiation type	Mo *K*α
μ (mm^−1^)	1.31
Crystal size (mm)	0.56 × 0.30 × 0.22

Data collection	
Diffractometer	Bruker D8 VENTURE
Absorption correction	Multi-scan (*SADABS*; Bruker, 2015 ▸)
*T*_min_, *T*_max_	0.626, 0.746
No. of measured, independent and observed [*I* > 2σ(*I*)] reflections	14360, 2800, 2597
*R*_int_	0.023
(sin θ/λ)_max_ (Å^−1^)	0.650

Refinement	
*R*[*F* ^2^ > 2σ(*F* ^2^)], *wR*(*F* ^2^), *S*	0.023, 0.055, 1.28
No. of reflections	2800
No. of parameters	136
H-atom treatment	H-atom parameters constrained
Δρ_max_, Δρ_min_ (e Å^−3^)	1.01, −0.59

Computer programs: *APEX3* and *SAINT* (Bruker, 2015 ▸), *SHELXT* (Sheldrick, 2015*a* ▸), *SHELXL* (Sheldrick, 2015*b* ▸), and *OLEX2* (Dolomanov *et al.*, 2009 ▸).

## supplementary crystallographic information

### Crystal data

**Table:**

(C_6_H_16_N)_2_[Sn(C_2_ClO_4_)_2_]	*F*(000) = 1160
*M**_r_* = 570.02	*D*_x_ = 1.558 Mg m^−^^3^
Monoclinic, *C*2/*c*	Mo *K*α radiation, λ = 0.71073 Å
*a* = 16.275 (8) Å	Cell parameters from 7290 reflections
*b* = 13.581 (6) Å	θ = 3.0–27.5°
*c* = 11.116 (4) Å	µ = 1.31 mm^−^^1^
β = 98.40 (3)°	*T* = 100 K
*V* = 2430.7 (18) Å^3^	Plate, clear light colourless
*Z* = 4	0.56 × 0.30 × 0.22 mm

### Data collection

**Table:**

Bruker D8 VENTURE diffractometer	2800 independent reflections
Radiation source: X-ray tube, Siemens KFF Mo 2K-90C	2597 reflections with *I* > 2σ(*I*)
TRIUMPH curved crystal monochromator	*R*_int_ = 0.023
Detector resolution: 1024 pixels mm^-1^	θ_max_ = 27.5°, θ_min_ = 3.0°
φ and ω scans'	*h* = −20→21
Absorption correction: multi-scan (*SADABS*; Bruker, 2015)	*k* = −17→17
*T*_min_ = 0.626, *T*_max_ = 0.746	*l* = −14→14
14360 measured reflections	

### Refinement

**Table:**

Refinement on *F*^2^	Primary atom site location: dual
Least-squares matrix: full	Hydrogen site location: inferred from neighbouring sites
*R*[*F*^2^ > 2σ(*F*^2^)] = 0.023	H-atom parameters constrained
*wR*(*F*^2^) = 0.055	*w* = 1/[σ^2^(*F*_o_^2^) + (0.011*P*)^2^ + 6.1885*P*] where *P* = (*F*_o_^2^ + 2*F*_c_^2^)/3
*S* = 1.28	(Δ/σ)_max_ = 0.002
2800 reflections	Δρ_max_ = 1.01 e Å^−^^3^
136 parameters	Δρ_min_ = −0.59 e Å^−^^3^
0 restraints	

### Special details

**Table:**

Geometry. All esds (except the esd in the dihedral angle between two l.s. planes) are estimated using the full covariance matrix. The cell esds are taken into account individually in the estimation of esds in distances, angles and torsion angles; correlations between esds in cell parameters are only used when they are defined by crystal symmetry. An approximate (isotropic) treatment of cell esds is used for estimating esds involving l.s. planes.

### Fractional atomic coordinates and isotropic or equivalent isotropic displacement parameters (Å^2^)

**Table:**

	*x*	*y*	*z*	*U*_iso_*/*U*_eq_	
Sn1	0.5000	0.67465 (2)	0.7500	0.01307 (6)	
Cl1	0.55290 (4)	0.56066 (4)	0.89962 (5)	0.02556 (12)	
O1	0.38232 (9)	0.69379 (11)	0.79635 (12)	0.0158 (3)	
O2	0.31479 (8)	0.77413 (11)	0.92602 (12)	0.0165 (3)	
O3	0.46345 (9)	0.85537 (11)	1.03026 (12)	0.0159 (3)	
O4	0.52285 (9)	0.78744 (10)	0.88059 (12)	0.0147 (3)	
C7	0.37835 (11)	0.75429 (14)	0.88463 (16)	0.0117 (3)	
C8	0.46144 (11)	0.80483 (14)	0.93878 (17)	0.0121 (4)	
N1	0.82801 (10)	0.66470 (12)	0.67738 (14)	0.0117 (3)	
H1A	0.8640	0.6618	0.6220	0.014*	
H1B	0.7824	0.6983	0.6421	0.014*	
C1	0.80130 (13)	0.56088 (15)	0.70045 (19)	0.0180 (4)	
H1	0.7608	0.5626	0.7599	0.022*	
C2	0.87571 (16)	0.49960 (18)	0.7530 (2)	0.0317 (6)	
H2A	0.8580	0.4317	0.7643	0.048*	
H2B	0.9003	0.5271	0.8316	0.048*	
H2C	0.9170	0.5004	0.6970	0.048*	
C3	0.75809 (16)	0.52045 (17)	0.5802 (2)	0.0268 (5)	
H3A	0.7113	0.5632	0.5490	0.040*	
H3B	0.7376	0.4539	0.5926	0.040*	
H3C	0.7975	0.5181	0.5215	0.040*	
C4	0.86854 (12)	0.72450 (16)	0.78415 (18)	0.0163 (4)	
H4	0.9228	0.6934	0.8176	0.020*	
C5	0.88477 (14)	0.82664 (16)	0.7363 (2)	0.0225 (4)	
H5A	0.9179	0.8209	0.6698	0.034*	
H5B	0.9151	0.8662	0.8020	0.034*	
H5C	0.8317	0.8586	0.7063	0.034*	
C6	0.81368 (14)	0.72851 (18)	0.88350 (19)	0.0231 (5)	
H6A	0.7582	0.7518	0.8491	0.035*	
H6B	0.8380	0.7738	0.9476	0.035*	
H6C	0.8094	0.6626	0.9179	0.035*	

### Atomic displacement parameters (Å^2^)

**Table:**

	*U*^11^	*U*^22^	*U*^33^	*U*^12^	*U*^13^	*U*^23^
Sn1	0.01120 (10)	0.01596 (10)	0.01279 (10)	0.000	0.00420 (6)	0.000
Cl1	0.0335 (3)	0.0221 (3)	0.0217 (2)	0.0028 (2)	0.0062 (2)	0.0098 (2)
O1	0.0111 (6)	0.0235 (7)	0.0134 (6)	−0.0049 (6)	0.0037 (5)	−0.0063 (6)
O2	0.0099 (6)	0.0262 (8)	0.0139 (7)	−0.0022 (6)	0.0035 (5)	−0.0046 (6)
O3	0.0147 (7)	0.0193 (7)	0.0130 (6)	−0.0013 (6)	0.0001 (5)	−0.0048 (5)
O4	0.0138 (7)	0.0166 (7)	0.0146 (7)	−0.0011 (5)	0.0056 (5)	−0.0016 (5)
C7	0.0112 (8)	0.0143 (9)	0.0095 (8)	−0.0013 (7)	0.0009 (7)	0.0026 (7)
C8	0.0088 (8)	0.0143 (9)	0.0125 (8)	−0.0005 (7)	−0.0008 (7)	0.0032 (7)
N1	0.0115 (7)	0.0134 (8)	0.0100 (7)	0.0003 (6)	0.0013 (6)	0.0021 (6)
C1	0.0200 (10)	0.0132 (9)	0.0211 (10)	−0.0016 (8)	0.0040 (8)	0.0049 (8)
C2	0.0323 (13)	0.0207 (11)	0.0403 (14)	0.0066 (10)	−0.0010 (11)	0.0129 (10)
C3	0.0327 (13)	0.0165 (10)	0.0312 (12)	−0.0058 (9)	0.0042 (10)	−0.0029 (9)
C4	0.0118 (9)	0.0227 (10)	0.0131 (9)	−0.0002 (8)	−0.0030 (7)	−0.0018 (8)
C5	0.0213 (11)	0.0208 (10)	0.0246 (11)	−0.0058 (9)	0.0006 (8)	−0.0019 (9)
C6	0.0250 (11)	0.0294 (12)	0.0144 (10)	0.0001 (9)	0.0016 (8)	−0.0015 (8)

### Geometric parameters (Å, º)

**Table:**

Sn1—Cl1	2.3422 (9)	C1—C3	1.519 (3)
Sn1—Cl1^i^	2.3422 (9)	C2—H2A	0.9800
Sn1—O1	2.0710 (16)	C2—H2B	0.9800
Sn1—O1^i^	2.0711 (16)	C2—H2C	0.9800
Sn1—O4^i^	2.1057 (15)	C3—H3A	0.9800
Sn1—O4	2.1058 (15)	C3—H3B	0.9800
O1—C7	1.289 (2)	C3—H3C	0.9800
O2—C7	1.222 (2)	C4—H4	1.0000
O3—C8	1.223 (2)	C4—C5	1.523 (3)
O4—C8	1.289 (2)	C4—C6	1.519 (3)
C7—C8	1.557 (3)	C5—H5A	0.9800
N1—H1A	0.9100	C5—H5B	0.9800
N1—H1B	0.9100	C5—H5C	0.9800
N1—C1	1.508 (3)	C6—H6A	0.9800
N1—C4	1.508 (2)	C6—H6B	0.9800
C1—H1	1.0000	C6—H6C	0.9800
C1—C2	1.514 (3)		
			
Cl1^i^—Sn1—Cl1	97.26 (4)	C2—C1—C3	112.4 (2)
O1—Sn1—Cl1	99.42 (5)	C3—C1—H1	109.0
O1—Sn1—Cl1^i^	90.13 (5)	C1—C2—H2A	109.5
O1^i^—Sn1—Cl1	90.13 (5)	C1—C2—H2B	109.5
O1^i^—Sn1—Cl1^i^	99.42 (5)	C1—C2—H2C	109.5
O1—Sn1—O1^i^	165.59 (8)	H2A—C2—H2B	109.5
O1—Sn1—O4	79.27 (6)	H2A—C2—H2C	109.5
O1—Sn1—O4^i^	90.21 (6)	H2B—C2—H2C	109.5
O1^i^—Sn1—O4^i^	79.27 (6)	C1—C3—H3A	109.5
O1^i^—Sn1—O4	90.21 (6)	C1—C3—H3B	109.5
O4^i^—Sn1—Cl1	168.50 (4)	C1—C3—H3C	109.5
O4—Sn1—Cl1^i^	168.49 (4)	H3A—C3—H3B	109.5
O4^i^—Sn1—Cl1^i^	88.95 (5)	H3A—C3—H3C	109.5
O4—Sn1—Cl1	88.95 (5)	H3B—C3—H3C	109.5
O4^i^—Sn1—O4	86.66 (8)	N1—C4—H4	109.0
C7—O1—Sn1	114.87 (12)	N1—C4—C5	107.09 (16)
C8—O4—Sn1	114.04 (12)	N1—C4—C6	110.83 (17)
O1—C7—C8	115.98 (16)	C5—C4—H4	109.0
O2—C7—O1	124.51 (18)	C6—C4—H4	109.0
O2—C7—C8	119.50 (17)	C6—C4—C5	111.83 (18)
O3—C8—O4	126.25 (18)	C4—C5—H5A	109.5
O3—C8—C7	119.01 (17)	C4—C5—H5B	109.5
O4—C8—C7	114.74 (16)	C4—C5—H5C	109.5
H1A—N1—H1B	107.1	H5A—C5—H5B	109.5
C1—N1—H1A	107.7	H5A—C5—H5C	109.5
C1—N1—H1B	107.7	H5B—C5—H5C	109.5
C4—N1—H1A	107.7	C4—C6—H6A	109.5
C4—N1—H1B	107.7	C4—C6—H6B	109.5
C4—N1—C1	118.25 (15)	C4—C6—H6C	109.5
N1—C1—H1	109.0	H6A—C6—H6B	109.5
N1—C1—C2	110.20 (18)	H6A—C6—H6C	109.5
N1—C1—C3	107.23 (16)	H6B—C6—H6C	109.5
C2—C1—H1	109.0		
			
Sn1—O1—C7—O2	−178.95 (15)	O2—C7—C8—O3	7.8 (3)
Sn1—O1—C7—C8	1.7 (2)	O2—C7—C8—O4	−172.75 (17)
Sn1—O4—C8—O3	168.18 (16)	C1—N1—C4—C5	−177.16 (17)
Sn1—O4—C8—C7	−11.23 (19)	C1—N1—C4—C6	−54.9 (2)
O1—C7—C8—O3	−172.83 (17)	C4—N1—C1—C2	−60.8 (2)
O1—C7—C8—O4	6.6 (2)	C4—N1—C1—C3	176.56 (17)

Symmetry code: (i) −*x*+1, *y*, −*z*+3/2.

### Hydrogen-bond geometry (Å, º)

**Table:**

*D*—H···*A*	*D*—H	H···*A*	*D*···*A*	*D*—H···*A*
N1—H1*A*···O3^ii^	0.91	2.05	2.943 (2)	167
N1—H1*B*···O2^i^	0.91	1.95	2.855 (2)	177

Symmetry codes: (i) −*x*+1, *y*, −*z*+3/2; (ii) *x*+1/2, −*y*+3/2, *z*−1/2.
